# Patient-Reported and Performance Outcomes Significantly Improved in Elderly Patients with Vestibular Impairment following Rehabilitation: A Retrospective Study

**DOI:** 10.1155/2018/5093501

**Published:** 2018-08-26

**Authors:** Daniel Héctor Verdecchia, Agustina Maria Monzón, Valentina Urbina Jaimes, Fernando Rocha Oliveira, Laércio da Silva Paiva, Tatiana Dias de Carvalho

**Affiliations:** ^1^Departamento de Ciencias de la Salud, Kinesiología y Fisiatría, Universidad Nacional de La Matanza, San Justo, Buenos Aires, Argentina; ^2^Área de Rehabilitación Vestibular, Universidad Maimónides, Ciudad Autónoma de Buenos Aires, Buenos Aires, Argentina; ^3^Departamento de Epidemiologia, Faculdade de Saúde Pública da Universidade de São Paulo, São Paulo, SP, Brazil; ^4^Laboratório de Epidemiologia e Análise de Dados, Departamento de Saúde da Coletividade, Faculdade de Medicina do ABC, Santo André, SP, Brazil

## Abstract

**Objective:**

To describe the results of a vestibular rehabilitation (VR) program in the timed up and go (TUG), gait speed (GS), and dizziness handicap inventory (DHI) scores for elderly vestibular patients in a developing country.

**Methods:**

Descriptive study with retrospective data collected from the clinical records of vestibular patients. The following information was recorded: sex, age, type of vestibular disorder, DHI score, and performance in TUG and GS, before and after participation in a VR program taking place from January 1 to August 30, 2017. The VR program consisted of 10 twice weekly sessions in the clinic and daily exercises at the patient's home. We used Student's *t*-test for paired and Wilcoxon's test according to the data distribution. The level of significance was 5%.

**Results:**

Data from 57 patients (49 females; 78 ± 5.8 years old) were used. There were statistically significant differences in TUG (12.52 versus 11.56), GS (0.81 versus 0.90 m/s), DHI total handicap (46 versus 24), physical (14 versus 8), emotional (14 versus 6), and functional (18 versus 12) domains.

**Conclusion:**

The functional outcome measures reported, including TUG, gait speed, and DHI, reflect statistically significant improvements in elderly patients after vestibular rehabilitation; the DHI improvements are clinically relevant.

## 1. Introduction

Patients with dysfunction in the vestibular system often complain of dizziness, balance impairments, and visual or gaze disturbances [1, 2]. In particular, dizziness is one of the most important single symptoms with a negative influence on well-being in old age [1]. While barriers to determining true prevalence and incidence exist due to differences in diagnosing and reporting, a previous survey showed that about 35% of people over 40 have experienced a vestibular disorder, a problem that can significantly impair the quality of life [2–4]. This dysfunction may be due to a disease-related pathology or trauma (surgical intervention) and can be located in the central (brain) or peripheral (inner ear) portions of the vestibular system.

One of the treatment options for vestibular disorders (unilateral and bilateral vestibular hypofunction and central or mixed vestibular disorders) is vestibular rehabilitation (VR). This consists of an individualized exercise program which has been developed to address the deficits identified during the physical therapy evaluation and which has been shown to be an effective treatment for patients with dizziness and balance disorders [5]. Currently, this program includes compensatory responses, adaptation for visual-vestibular interaction, substitution and postural control exercises, fall prevention, (re)conditioning activities, and functional/occupational retraining [6].

The objective of the VR program is to encourage compensation after peripheral and central vestibular disorders, thus reducing symptoms of dizziness and vertigo and the risk of falls, while increasing confidence in equilibrium and encouraging the return to activities of daily living [5, 7]. The efficacy of vestibular rehabilitation can be evaluated using performance-based outcome measures such as gait speed (GS), timed up and go (TUG) [8], and patient-reported outcome measures such as the dizziness handicap inventory (DHI) [9–11]. The outcome measures are simple, easy, and quick to administer in a clinical setting. There is moderate to strong evidence to support the use of vestibular rehabilitation for persons with peripheral unilateral vestibular disorders [5, 12]. Information is still lacking, however, on the population that suffers from peripheral and central vestibular disorders and the effects of VR programs, especially in developing countries.

Another major problem in elderly populations, one which is not a hypofunction but rather a mechanical disorder, is benign paroxysmal positional vertigo (BPPV). This is believed to be one of the most common causes of vertigo [13] and can be present at any age but is most common among people in their 6th and 7th decades [14]. BPPV is caused by free otoconia dislodged from the utricular macula that have entered the semicircular canal, where they provoke an inappropriate flow of endolymph whenever the head is rotated in the plane of the affected canal [15]. Residual dizziness (RD) is a common condition in patients with idiopathic BPPV, which starts in the first episode, even after quick resolution with repositioning maneuvers. Early treatment of BPPV is advisable for preventing RD, especially in anxious and elderly patients [16].

For BPPV, there is considerable evidence to support the use of repositioning maneuvers at the outset and also to show that vestibular rehabilitation should be incorporated in the long term as a preventative measure or to promote functional recovery or both [6]. A recent clinical practice guideline concluded that a clinician may offer VR in the treatment of BPPV for patients with additional impairments and that this can result in prevention of falls and an improved return of natural balance function [17]. A recent systematic review [18] found that there is an urgent need for more research conducted in low-middle-income environments. Here in our country, we have not found any studies evaluating VR or describing its impact on functional test results (TUG and GS). The objective of this study, then, is to describe the outcomes of a vestibular rehabilitation program on the timed up and go, gait speed, and dizziness handicap inventory scores for elderly vestibular patients in a developing country.

## 2. Methods

### 2.1. Study Design and Population

This is a descriptive study with retrospective data collected from the clinical records of vestibular patients aged 65 years or older at a vestibular rehabilitation clinic located in an urban middle-income environment. The following information was recorded: sex, age, type of vestibular disorder, DHI score, and performance in TUG and GS, before and after their participation in a vestibular rehabilitation program taking place from January 1 to August 30, 2017.

Patients were referred by otolaryngologists. In those cases where the prescription did not contain a specific diagnosis, an experienced physiotherapist evaluated each subject by means of a battery of clinical oculomotor (ocular alignment and tests of Skew, ocular range of motion, smooth pursuit, volitional saccades, and vergence) and vestibular tests (vestibulo-ocular reflex cancellation test (VORc), head impulse test, head shaking test, or mastoid vibration test), thus ensuring that the disorder was primarily of the vestibular origin. The patients who gave positive results in the clinical vestibular tests were included in the study [19].

Incomplete records, failure to complete the rehabilitation program and other neurologic conditions (Parkinson's disease, multiple sclerosis, stroke, and others) were the criteria for exclusion. People without a specific diagnosis who had central signs during the physiotherapist's evaluation were referred to a neurologist, and patients who only suffered from BPPV without residual symptoms were not included in a VR program, thus no data from functional tests were found in the clinical history of these two groups of patients (incomplete records).

Both the rehabilitation clinic and the patients signed consent forms authorizing all procedures, which was carried out in accordance with the Code of Ethics of the World Medical Association (Declaration of Helsinki) and authorized by the local ethics committee.

### 2.2. Vestibular Rehabilitation Program

The VR program consisted of 10 twice weekly sessions in the rehabilitation clinic and daily exercises at the patient's home. It was based on the treatment protocol published in Binetti et al. [7] and Verdecchia et al. [20], with adaptation exercises and/or habituation and/or substitution exercises determined and adapted in accordance with an assessment of the patient. Subjects received vestibular and balance rehabilitation provided by three physical therapists with specialized training in the treatment of balance and vestibular disorders.

The vestibulo-ocular and oculomotor exercises included X1 and X2 viewing paradigm (near and far) with periods of stimulation 1 to 2 minutes long (3–5 times daily for a total of 20–40 minutes per day). Oculomotor training for patients with bilateral vestibular hypofunction included two cards exercises (visualization of 2 targets) and remembered target (1 target), designed to challenge saccadic movements and gaze shifting [12]. The vestibulospinal exercises included static balance with progressive reduction of the support base, eyes open and closed, and firm to soft surface. The dynamic balance and gait tasks included walking with head and body turns, velocity changes, and walking with progressively narrower base of support.

Patients who suffered from BPPV with residual symptoms (imbalance and dizziness) were treated with repositioning maneuvers before they were included in a customized VR program based on a functional evaluation of each patient [3, 17]. Some non-BPPV patients with dizziness provoked by their own head or body movement were prescribed habituation exercises based on the results of the 16 movements in the motion sensitivity quotient (MSQ) [21]. The movements chosen for habituation were performed as follows: four repetitions four times a day, until the exercises did not generate any symptoms for 48 h, at which time they were suspended.

In addition, all the subjects performed adaptation exercises for the vestibulo-ocular and vestibulospinal reflexes, three to five times a day in their homes, for a total stimulus time of 20 to 40 minutes daily.

### 2.3. Timed Up and Go

Subjects were asked to complete 3 trials of the TUG. For this, they were given verbal instructions to stand up from a seated position on a chair, walk 3 m as quickly and as safely as possible, cross a line marked on the floor, turn around, walk back, and sit down. The stopwatch was started on the word “go” and stopped when the subject'is back came into contact with the chairback after sitting down. The TUG time was measured in seconds (sec). One practice trial was given, and the score discarded. Two scored trials were then performed, and the average of these scores was used for this study [22]. Those subjects who used an assistive device when walking in the community were asked to use that device [23]. Whitney el al. [24] found that a TUG cutoff of 11.1 seconds appeared to display the best balance between sensitivity (80%) and predictive positive value (46%) in patients with vestibular dysfunction. They concluded that the sensitivity of the TUG test for fall prediction was 80%, and the specificity was 56% in patients who scored greater than 11.1 seconds on the test. People who scored greater than 11.1 seconds on the TUG were 5 times (*p*=0.001) more likely to have reported a fall in the previous 6 months.

### 2.4. Gait Speed

To evaluate the usual gait speed, we used the timed 10-meter walk test. Each patient was instructed to walk 10 meters at a comfortable and normal pace (3 trials). Only the middle 6 meters section was timed to eliminate the effects of acceleration and deceleration. A certified examiner started the timing when the subject's first foot crossed the “start” line and stopped timing when his/her last foot crossed the “finish” line. Walking speed (in meter/second, or m/s) was calculated as walking distance (20 ft = 6.10 m) divided by time (in seconds), and the average of the 3 trials was registered [25, 26]. Older persons with gait speed slower than 1 m/s (equal to or more than 6 seconds to walk 6 m) should be considered at high risk of adverse health outcomes [27].

### 2.5. Dizziness Handicap Inventory

The DHI is a 25-item tool used to help the patient rate their self-perception of handicap from dizziness [9]. It is subdivided into functional (36 points), emotional (36 points), and physical domains (28 points) and ranges from zero (no perceived handicap) to 100 (the maximum perceived handicap) [10]. We decided to use the Argentine version of this questionnaire [28], which is a reliable and valid tool for quantifying self-perceived handicap resulting from vertigo, dizziness, or unsteadiness and has high internal consistency (*α* = 0.87) and very high test-retest reliability for the total DHI score (intraclass correlation coefficient: 0.98) and its domains.

### 2.6. Statistical Analysis

We have carried out a descriptive analysis of the data. The characteristics of the population are presented by absolute and relative frequency, and the quantitative variables are presented by measures of central tendency and dispersion in accordance with the normality test (Shapiro–Wilk test). To analyze the tests and perception of impairment before and after the rehabilitation program, we used Student's *t*-test for paired and Wilcoxon's test in accordance with the data distribution. The level of significance was 5%. The statistical program used was the Stata 12.0 version.

## 3. Results

We have evaluated 76 clinical records. Of these, 19 were excluded because of incomplete information and failure to complete the rehabilitation program. Data from 57 patients (49 females; 78 ± 5.8 years old) with vestibular disorders were used. Unilateral vestibular hypofunction and BPPV had a higher prevalence with 29 (50.88%) and 12 (21.05%) of the cases, respectively.  Table 1 presents the population characteristics.

**Figure 1:**
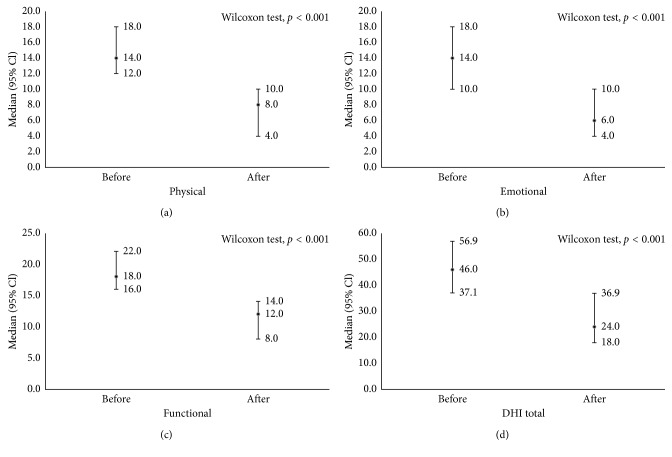
DHI total and respective domains and pre- and postvestibular rehabilitation program.

The median improvement in the total DHI score was about 22 points after VR (a change >18 points meets the minimally clinically important difference (MCID) for the vestibular population). The median total DHI score before VR was 46 (CI 95% 37.13–56.86) and after, 24 (CI 95% 18.00–36.86), demonstrating improvements after VR (Wilcoxon signed rank, *p* < 0.001). In the analyses of pre- and post-VR median DHI points, a decrease and a consequent improvement were observed in all domains: emotional (pre-VR 14 (CI 95% 10.00–18.00), post-VR 6 (CI 95% 4.00–10.00), and Wilcoxon-signed rank test *p* < 0.0001), functional (pre-VR 18 (CI 95% 16.00–20.00), post-VR 12 (CI 95% 8.00–14.00), and Wilcoxon-signed rank test *p* < 0.0001) and physical (pre-VR 14 (CI 95% 12.00–18.00), post-VR 8 (CI 95% 4.00–10.00), and Wilcoxon-signed rank test *p* < 0.0001).  Figure 1 shows the results of total DHI and its respective domains, pre- and postvestibular rehabilitation program.

The mean gait speed increased by 0.09 m/sec after VR. Mean values obtained before and after VR were 0.81 m/sec (SD: 0.21) *y* and 0.90 m/sec (SD: 0.19), respectively (Student's *t*-test, *p* < 0.001).

Following VR, the median TUG decreased by 0.96 sec. In the analysis of pre- and post-VR TUG results, the medians were 12.52 (CI 95% 11.33–13.15) and 11.56 (CI 95% 10.58–12.08), respectively (Wilcoxon-signed rank test, *p* < 0.001).

## 4. Discussion

Our study shows that there were statistically significant differences in timed up and go, gait speed and dizziness handicap inventory values after a VR program. Patients were predominantly female, and the most common diagnosis was unilateral vestibular hypofunction.

This type of disorder affects both sexes, and since there is evidence suggesting that the patient's sex may not impact rehabilitation outcomes, a VR program can be offered to males and females with the expectation of similar outcomes [12].

While Jung et al. [29] and Moreira Bittar et al. [30] have noted that age is not a significant factor in the response rate to vestibular exercises, Smith-Wheelock et al. [31] have reported that older patients required modifications in therapy due to increased risk for falls. In the same study, more of the patients in the older group required supervised therapy compared to the younger group. However, the only significant difference noted for older than 65 years was in the length of time required to maximize the benefit from therapy.

As for diagnoses, our findings coincide with a previous study [32]. Vestibular rehabilitation is also indicated for the most common type of vestibular disorders in this study: unilateral vestibular hypofunction (regardless of whether it is on the left or right side). Bayat et al. [33] found no significant differences in VR outcomes between men and women or those with right- or left-side lesions (*p* > 0.05). Tee and Chee [34] reported that individuals with stable unilateral peripheral vestibular loss with incomplete central compensation benefited the most from a VR program, but the treatment strategies are different for unilateral and bilateral dysfunction [5].

BPPV is the most widely recognized vestibular disorder [5] and is characterized by vertigo of short duration after a change in the position of an individual's head with respect to gravity and with symptoms typically lasting less than one minute [5, 35]. Our study included 12 patients with unilateral posterior BPPV who presented with residual dizziness and unsteadiness after successful canalith repositioning procedures (CRPs). While there is reason to believe that habituation exercises cannot replace CRPs in the initial treatment of BPPV, adaptation and habituation exercises can nevertheless serve as adjuvant therapy for selected patients with BPPV [17]. BPPV can be associated with significant residual complaints of generalized dizziness (abnormal motion sensitivities not associated with provocation of nystagmus) and definable abnormal postural control with heightened fall risk even after CRP has successfully resolved paroxysmal positional nystagmus [36, 37]. There is a statistically significant increased risk for persistent postural abnormalities in the elderly in general where multifactorial comorbid impairments may be present [38]. A randomized controlled trial (RCT) showed that subjects with BPPV who were treated with CRP and additional VR exercises had significantly improved measures of overall gait stability when compared with those who had received isolated CRP (Epley maneuver) for their BPPV [39].

A limitation of our study is that the results were obtained from patients with different disorders and have not been classified accordingly. However, there is promising evidence that suggests that VR improves symptoms and can reduce falls in persons with a variety of vestibular conditions [5]. In the present study, results show statistically significant differences in TUG, GS, and DHI values, indicating improvement in the patients' performance and reported outcomes after a VR program, thus demonstrating the effectiveness of such intervention even in a developing country.

Our results for TUG are different from the study of Gill-Body et al. [40], whose values were greater than ours. Their results were unilateral vestibular hypofunction 19.5 (5.72), range 12.67–39.0, *n* = 34; bilateral vestibular hypofunction 23.33 (11.66), range 12.74–52.01, *n* = 44. This difference was probably due to the fact that they used a modified version of the TUG.

After the VR program, an increase was observed in GS and a decrease in TUG scores. This corroborated the previous studies [16, 41, 42] demonstrating that after a VR program, patients with vestibular disorders usually walk faster [43, 44]. Gait speed has been considered an important indicator of patient health, regardless of the diagnosis [45]. Indeed, it has been observed in another study that older adults with impaired semicircular canal function had slower gait compared to adults with intact function [46]. However, there is no published evidence of minimal detectable change (MDC) in the gait speed tests (GSTs) or in the TUG for vestibular patients. Although a number of authors have published studies with MDC values in other patient populations and these values range from 0.08 to 0.19 m/sec in GS [47–49] and from 1.10 to 4.00 sec in TUG [50–53], these populations were different from vestibular patients and consequently we can't be sure that they are suitable for our purposes here. Studies on vestibular populations are necessary. In our study, the difference found in the pre- and post-RV TUG scores was statistically significant but failed to reach the lowest MDCs already published with reference to other populations. The TUG values in our study were similar to those found in patients with mild DHI (TUG 12 ± 3 sec) and moderate DHI (TUG 11 ± 4 sec) in Whitney et al. [10].

With regard to patients' perception of impairment at the completion of the VR program, there were statistically significant differences in total and functional DHI as well as in the emotional and physical domains. Whitney et al. [10] categorized the DHI scores into mild handicap (0–30), moderate handicap (31–60), and severe handicap (61–100); therefore, higher scores on the DHI indicate greater handicap. Using this classification, our results indicate that patients progressed from moderate to mild handicap. The TUG and gait speed values reported by Whitney et al. [10] were similar to ours (TUG and GS in mild DHI: 12 ± 3 and 1.02 ± 0.2; in moderate DHI: 11 ± 4 and 1.04 ± 0.2; in severe DHI: 14 ± 5 and 0.9 ± 0.2). This study was also similar to ours in that the functional test results from a group of 85 patients with vestibular disorders (peripheral and central) were analyzed without being broken down into subgroups. The reduction we found in the posttreatment score was superior to the 18-point minimum clinically important difference (MCID) published by Jacobson et al. [9]. These results of DHI coincide with the study of Humphriss et al. [32] and also with one study carried out in our country in which statistically significant reductions in DHI values were observed after VR [20]. These authors analyzed differences in the self-perception of impairment, the risk of falls, and gaze stability in patients with chronic unilateral vestibular hypofunction before and after VR with complementary Wii® therapy. Unlike the present study, there was no evaluation of the TUG or the GS; however, they also found an improvement in DHI scores.

Taken together, our results point to the importance of VR programs, regardless of the diagnosis of vestibular disorders. The rehabilitation program in this study consisted of exercises of adaptation and/or habituation and/or substitution, depending on each patient, twice a week in the rehabilitation clinic, and daily at the patient's home [7, 20]. Clinical practice guidelines for vestibular hypofunction suggest that the number of visits may need to be varied in accordance with comorbidities that can affect movement and psychological functioning [12].

The frequency, duration of optimal treatment, and total number of visits for the best results from physical therapy are as yet unknown. Knowledge on the effects of VR programs in developing countries is still incipient. In our country, this is one of the few studies carried out so far that evaluates the benefits of this type of care and the first to describe the effects of a VR program on TUG and gait speed in older people. Further studies are needed to observe the effects of this treatment in other Latin American countries in order to facilitate the dissemination of VR in the region and to compare performance in functional tests and self-perception of impairment in these countries. It should be noted that the results of this study were reported after the completion of a VR program in South America, which shows that in developing countries there are physical therapists who have been trained to carry out vestibular evaluation and exercise protocols. The cultural, economic, and social differences among environments with low, middle, and high incomes and their impact on the outcomes of VR have to date not been reported. We may hypothesize that these differences could change the results of a rehabilitation process and must be studied.

This study has some other limitations. The sample was one of convenience, and while it represented a wide range in function and cognition, all of the participants were from an urban area and may not be representative of all older people in the same area. We did not have information on the patients' socioeconomics and educational level, factors which may have affected performance. The testing sessions were not conducted in conditions of privacy, so the influence of other older adults or staff in the room may have affected performance. Although there were more females than males in our study, we do not believe that this influenced our results since a recent guideline [12] affirms that a VR program should be offered to males and females with the expectation of similar outcomes. Another limitation is the retrospective design. However, the patient records were all from a specialized VR clinic which used a uniform procedure for the evaluation and treatment of all its patients, and the outcome measures were obtained by the same experienced physical therapist, that is, the therapist who took the measurement for a specific patient was always the same, thus increasing the reliability of the measurement.

Some of our participants had a nonspecific diagnosis (such as dizziness or vertiginous syndrome). In these cases, an experienced physiotherapist assessed each subject with clinical vestibular tests [22].

Randomized controlled trial evidence for the efficacy of customized VR is available elsewhere, as already described. The present study demonstrates the effectiveness of VR where it has been successfully applied in a common clinical context in an urban middle-income environment. The study results are relevant to function and quality of life in elderly patients; yet, more research is needed to assess the effects of vestibular rehabilitation on quality of life and frequency of falls.

## 5. Conclusion

The functional outcome measures reported, including TUG, gait speed, and DHI, reflect statistically significant improvements in elderly patients after vestibular rehabilitation; the DHI improvements are clinically relevant.

## Figures and Tables

**Table 1:** Population characteristics.

Variables	*n*	%
*Gender*		
Female	49	85.96
Male	8	14.04
*Diagnoses*		
Unilateral vestibular hypofunction (UVH)	29	50.88
Benign paroxysmal positional vertigo (BPPV)	12	21.05
Nonspecific diagnosis	6	10.53
Multisensory dizziness syndrome	5	8.78
Bilateral vestibular hypofunction	3	5.26
Mixed vestibular disorder	1	1.75
Central vestibulopathy	1	1.75
*Classification of diagnosis*		
Peripheral	50	87.72
Central	6	10.53
Mixed	1	1.75

## Data Availability

The data used to support the findings of this study are available from the corresponding author upon request.
